# Diagnostic and prognostic ability of salivary MMP-9 for oral squamous cell carcinoma: A pre-/post-surgery case and matched control study

**DOI:** 10.1371/journal.pone.0248167

**Published:** 2021-03-18

**Authors:** Yoo-Jin Shin, Huong Vu, Jong-Ho Lee, Hyun-Duck Kim

**Affiliations:** 1 Department of Preventive and Social Dentistry, School of Dentistry, Seoul National University, Seoul, Korea; 2 Oral Cancer Center and Department of Oral and Maxillofacial Surgery, Seoul National University Dental Hospital, Seoul, Korea; 3 Dental Research Institute, Seoul National University, Seoul, Korea; University of Oslo, NORWAY

## Abstract

Over 90% of oral cancers are oral squamous cell carcinoma (OSCC). Hitherto, early detection marker for OSCC has not been available. Hence, this study aimed to evaluate the diagnostic and prognostic ability of salivary matrix-metalloproteinase-9 (MMP-9) and 8-hydroxydeoxyguanosine (8-OHdG) for OSCC. Total of 318 participants with 106 cases and 212 controls were included: OSCC cases were from Seoul National University Dental Hospital and age, sex, and smoking matched controls were from Yangpyeong cohort. Unstimulated saliva was collected to determine MMP-9 and 8-OHdG using sensitive enzyme-linked immunosorbent assay. Multivariable linear regression and analysis of covariance (ANCOVA) were applied to evaluate the adjusted association of markers with OSCC. Wilcoxon sign rank sum test and Friedman test for median were applied to evaluate follow-up level of MMP-9 after surgery. Receiver operating characteristic curve was obtained for diagnostic ability. Salivary MMP-9 was associated with OSCC (ANCOVA and multivariable linear regression, p<0.05), while 8-OHdG was not. The diagnostic ability of MMP-9 was area under curve of 0.96 (100% specificity and 89.6% sensitivity, p<0.001). MMP-9 decreased dramatically after tumor surgery (p<0.05). Salivary MMP-9 could be a critical diagnostic and prognostic marker for OSCC.

## Introduction

Head and neck cancer (HNC) comprising of oral cavity, oropharynx, and larynx is the sixth most common cancer worldwide, with a high morbidity rate, and a 5-year survival rate of only 50% [1,2]. Over 90% of oral cancers are oral squamous cell carcinoma (OSCC) that arises from the squamous epithelium which are often seen on the 5^th^-6^th^ decade of life [3]. Even though OSCC is in the most accessible area for visual examination, there is no early warning signs and symptoms for patients until the later stage. Hence it was diagnosed mostly in advanced stages and the survival rate was low [4,5]. This suggests that sensitive and specific biomarkers are likely to be valuable in screening high-risk patients for earlier diagnosis, which guarantees better prognosis and better prognosis and decrease morbidity and mortality rate.

One important hallmark of cancer progression is the degradation of the extracellular matrix (ECM), which allows cancer cells to invade the surrounding tissue. Matrix-metalloproteinase-9 (MMP-9) is known to efficiently degrade the components of the ECM and basement membrane [6]. Another molecular marker known for initiation and promotion of carcinogenesis is a DNA damage marker, an oxidative stress-related molecule, 8-hydroxy-2-deoxyguanosine (8-OHdG) [7].

There are some original articles for salivary MMP-9 and three original articles for salivary 8-OHdG, that utilized salivary analysis to evaluate the association with OSCC. However, the association between salivary MMP-9 and OSCC has not been clear. For salivary MMP-9, one study focused on 19 tongue cancer patients [8] while the other one was a case-control study with total 50 participants [9]. Both of them did not show significant association after adjustments of covariates. The other two articles also revealed higher salivary MMP-9 in OSCC patients as compared to controls but no adjustments were done [10,11]. For 8-OHdG, three studies focused more on pre-cancerous lesions like oral lichen planus (OLP), oral leukoplakia or oral submucous fibrosis [12–14]. All studies had limitations of having small number of participants and no/insufficient adjustments. Hitherto, the association of salivary MMP-9 and 8-OHdG with OSCC is still controversial and its prognostic effect has not been clarified yet. Therefore, this study aimed to evaluate the association of salivary MMP-9 and 8-OHdG among large OSCC patients and age, sex, smoking matched non-OSCC controls with adjustments of covariates, and to investigate the prognostic and screening ability of these biomarkers for OSCC.

## Materials and methods

### Ethical consideration and study design

This study was approved by the Institutional Review Board for Human Subjects of the Seoul National Dental Hospital for cancer cases (CRI15017) and for Human Subjects of Seoul National University School of Dentistry for cohort controls (S-020060000). It has been confirmed that all research was performed in accordance with relevant guidelines and regulations. All participants voluntarily provided a written informed consent statement after hearing out full explanation of the purpose of this study. This case-control and follow-up study follows the Strengthening the Reporting of Observational studies in Epidemiology (STROBE) guidelines (S1 Checklist).

### Sample size estimation

The sample size of this study was estimated using paired-T test power analysis under the condition of type I error of 0.05 and type II error of 0.2. The difference in MMP-9 between operation and 3months after surgery groups from the pilot data was 200 ng/ml with standard deviation of 550 ng/ml. The calculated sample size for cases was 52. In consideration of attrition rate of 50%, total sample size for cases was decided as 104. The ratio between cases and controls was 1:2 and total sample size was decided 312 with 104 for cases and 208 for controls.

### Selection of participants

OSCC cases for this study were 106 voluntary participants (62 males, 44 females) who visited the outpatient clinic of Department of Oral and Maxillofacial Surgery in Seoul National University Dental Hospital (SNUDH) from January, 2015 until December, 2018. The inclusion criteria for cases with OSCC as final diagnosis were as follows: 1) agreed to be participants voluntarily, 2) enough saliva sample (more than 30 ul of saliva sample) for the analysis and validation 3) no history of chemotherapy and/or radiotherapy, 4) no missing information of covariates included in this study, 5) no pregnancy, 6) no auto-immune disorders. Controls suitable to inclusion criteria were randomly selected by matching for age, sex, and smoking of cancer cases from 3090 baseline participants in the Yangpyeong cardiovascular and periodontal health cohort. This cohort was a part of Korean Genome Epidemiologic Study (KoGES) supported by the Korea Centers for Disease Control and Prevention from 2010 to 2014. This cohort participants were diagnosed as oral cancer negative according to the detecting guideline of oral cancer by NCI, NIH [15]. Out of the enrolled 156 OSCC patients with OSCC as final diagnosis, only 106 cases satisfied the inclusion criteria for MMP-9 and 78 cases for 8-OHdG due to the insufficient salivary sample for the analysis. Two controls were matched to one case according to age, sex and smoking. Finally, total of 318 participants (106 cases and 212 controls) were included for final analyses of MMP-9 and total of 234 participants (78 cases and 156 controls) for 8-OHdG. All participants provided demographic information such as age and sex, health-related behavioral variables and dental/medical history of oral/systemic diseases.

### Assessment of oral squamous cell carcinoma

Oral examination was done on the first visit by a single oral and maxillofacial surgeon (JH Lee) followed by incisional biopsy after careful clinical and radiographic observation of the suspected lesion. Final diagnosis of OSCC was confirmed by the two departments in SNUDH: by the oral pathologists from the Department of Oral and Maxillofacial Pathology through the examination of the specimen histologically, and by the oral radiologists from the Department of Oral and Maxillofacial Radiology through evaluation of the tumor type and location using panoramic radiograph, enhanced computed tomography (CT), magnetic resonance imaging (MRI) and positron emission tomography–computed tomography (PET-CT). After reviewing the two reports, patients subjected to undergo surgery were included in this study. After surgery, information on the location of tumor and tumor-node-metastases (TNM) staging [16] were obtained from the gross biopsy of tumor by using the International Classification of Diseases for Oncology [ICD-0] codes C02.0-C06.9. As for TNM staging, our study included carcinoma in situ which was noted as stage 0 [17].

### Saliva sampling

Unstimulated whole saliva was collected from each patient after informing the patient on the collection method following the previous protocol [18]. Patients were recommended not to eat, drink nor brush their teeth one hour before the sample collection. Patients passively drooled or spitted for 10 minutes in a relaxed sitting position into the 50 ml conical tube. Secretion rate was recorded then vials were centrifuged in 2,600 rpm for 15 minutes with 4°C temperature, the supernatants were aliquoted by 1 ml into an autoclaved 1.5 ml Eppendorf tube. Aliquoted vials were then stored into -80°C deep freezer. Needed saliva samples were thawed to be used in the analyses. The same saliva collection method was used in controls.

### Assessment of MMP-9 and 8-OHdG

Salivary biomarkers, MMP-9 and 8-OHdG, were evaluated by sensitive enzyme-linked immunosorbent assay (ELISA) kit. Quantikine® human MMP‐9 immunoassay (R&D Systems, Inc., Minneapolis, MN) was used for salivary MMP-9 and 8-OHdG Check (Japan Institute for the Control of Aging, Shizuoka, Japan) was used for salivary 8-OHdG. All tests followed the manufacturer’s instruction guidelines. Saliva samples were diluted using serial dilution method with reagent diluent provided by manufacture (1, 1/2, 1/4, 1/8, 1/16, 1/32) and values of MMP-9 and 8-OHdG in diluted saliva samples were calculated from each standard curve. We decided the standard dilution rate that falls in the range between 1.25 to 10 ng/ml for MMP-9 and 0.125 to 10 ng/mL for 8-OHdG.

### Assessment of confounders

Socio-demographic information such as age, sex and education, health-related behavioral variables such as smoking, drinking and physical exercise, and health status such as periodontitis, hypertension, diabetes, obesity and hypercholesterolemia were considered as confounders for inflammatory biomarkers including MMP-9 and 8-OHdG. Socio-demographic information was obtained from face-to-face interview. Health status was obtained from oral and physical examination by dentists and physicians and the serum laboratory test results.

Variables were dichotomized, as for education: until middle school and high school or higher; for smoking: smoker and non-smoker; for drinking: drinker and non-drinker; for physical activity: doing physical activity on a daily basis for at least 30 minutes in any form of exercise and non-active. Obesity was dichotomized using Body mass index (BMI) which was calculated as weight (kg) divided by the square of height (m^2^) as: no, < 25.0 kg/m^2^ and yes, ≥25.0 kg/m^2^ and above [19]. For biochemical variables, 12-h fasting blood samples were drawn at recruitment. Serum biomarkers included in this study were fasting plasma glucose (FPG) (mg/dl) and fasting total cholesterol (FTC) (mg/dl). Diabetes was validated by categorizing FPG into 2 groups according to the diagnostic criteria of American Diabetes Association [20]: no = FPG<126mg/dl and yes = FPG ≥126mg/dl, taking insulin shots or taking anti-diabetic medication and/or diagnosed by the physician. Hypercholesterolemia was validated by categorizing into 2 groups according to the criteria of National Cholesterol Education Program Adult Treatment Panel III (NCEP-ATP III) [21]: no = FTC<240 mg/dl and yes = FTC≥240mg/dl, or taking anti-cholesterol medication. The systolic blood pressure (SBP) and diastolic blood pressure (DBP) (mmHg) of the subjects were measured in the sitting position using a mercury manometer two times with 15-minutes intervals, and the mean value of two trials was used in the analysis. Hypertension was evaluated by categorizing into 2 groups according to the criteria of Joint National Committee on the Prevention, Detection, Evaluation, and Treatment of High Blood Pressure (JNC) 7th, 2004: no = SBP<140 mmHg or DBP<90mmHg and yes = SBP≥140mmHg or DBP≥ 90mmHg or taking anti-hypertensive medication [22].

For periodontitis, radiographic alveolar bone loss (RABL) as the surrogate of clinical attachment loss (CAL) was assessed by a trained dentist (YJ Shin) using the panoramic radiograph (Orthopantomograph OP100, GE Healthcare, Finland). RABL was measured from the cemento-enamel junction (CEJ) of the tooth up to the highest point of the proximal alveolar bone crest on the mesial and distal of the remaining natural teeth except the 3^rd^ molars [23]. Following the guidelines from the 5^th^ European workshop in Periodontology, Periodontitis was classified into 3 which were as follows: “Normal” if there is presence of proximal bone loss < 3mm or ≥3mm in < 2 non-adjacent teeth, “Incipient” if there is presence of proximal bone loss of ≥3mm in ≥2 non-adjacent teeth, “Severe” if there is presence of proximal bone loss of ≥5mm in ≥30% of teeth present [24]. In this study, incipient and severe periodontitis were regarded as periodontitis (“yes”).

### Statistical analysis

Baseline characteristics of the participants were shown as mean and standard deviations (SD) for continuous variable and number and percentages for categorical variables. In comparison, T-test was applied for continuous variable and chi-square test for categorical variables (Table 1). We applied correlation analysis for the relationship between MMP-9 and 8-OHdG.

**Table 1:** Characteristics of variables according to cancer case and control.

	MMP-9* (N = 318)			8-OHdG^†^ (N = 234)		
Variable	Cancer case (n = 106)	Control (n = 212)	*p*-value	Cancer case (n = 78)	Control (n = 156)	*p*-value
**Age, mean±SD**	63.14 ± 9.7	63.09 ± 9.7	0.964	63.62 ± 9.2	63.62 ± 9.1	0.996
**Sex, n (%)**			1.0			1.0
Male	62 (58.5)	124 (58.5)		45 (57.7)	90 (57.7)	
Female	44 (41.5)	88 (41.5)		33 (42.3)	66 (42.3)	
**Smoking**^**§**^**, n (%)**			1.0			1.0
No	64 (60.4)	128 (60.4)		48 (61.5)	96 (61.5)	
Yes	42 (39.6)	84 (39.6)		30 (38.5)	60 (38.5)	
**Alcohol intake**^**║**^**, n (%)**			**0.001**			**0.001**
No	62 (58.5)	80 (37.7)		46 (59.0)	57 (36.5)	
Yes	44 (41.5)	132 (62.3)		32 (41.0)	99 (63.5)	
**Education level, n (%)**			**<0.001**			**<0.001**
Middle school	34 (32.1)	129 (60.8)		28 (35.9)	101 (64.7)	
High school or higher	72 (67.9)	83 (39.2)		50 (64.1)	55 (35.3)	
**Physical activity**^**¶**^**, n (%)**			**0.006**			0.083
No	79 (74.5)	124 (58.2)		57 (73.1)	96 (61.5)	
Yes	27 (25.5)	88 (41.5)		21 (26.9)	60 (38.5)	
**Periodontitis**^**‡**^**, n (%)**			**<0.001**			**0.001**
No	6 (5.7)	51 (24.1)		5 (6.4)	37 (23.7)	
Yes	100 (94.3)	161 (75.9)		73 (93.6)	119 (76.3)	
**Obesity**^**#**^**, n (%)**			0.614			0.475
No	73 (68.9)	139 (65.6)		52 (66.7)	96 (61.5)	
Yes	33 (31.1)	73 (34.4)		26 (33.3)	60 (38.5)	
Diabetes^**^, n (%)			**0.011**			0.132
No	83 (78.3)	189 (89.2)		61 (78.2)	135 (86.5)	
Yes	23 (21.7)	23 (10.8)		17 (21.8)	21 (13.5)	
**Hypertension**^††^**, n (%)**			0.225			0.470
No	37 (34.9)	90 (42.5)		25 (32.1)	59 (37.8)	
Yes	69 (65.1)	122 (57.5)		53 (67.9)	97 (62.2)	
**Hypercholesterolemia**^**‡‡**^**,n(%)**			**0.003**			**0.019**
No	87 (82.1)	198 (93.4)		65 (83.3)	146 (93.6)	
Yes	19 (17.9)	14 (6.6)		13 (16.7)	10 (6.4)	

The ratio between cancer cases and controls matched for age, sex and smoking was two.

*MMP-9: Matrix metalloproteinase-9.

^†^8-OHdG: 8-hydroxydeoxyguanosine.

**Fig 1:**
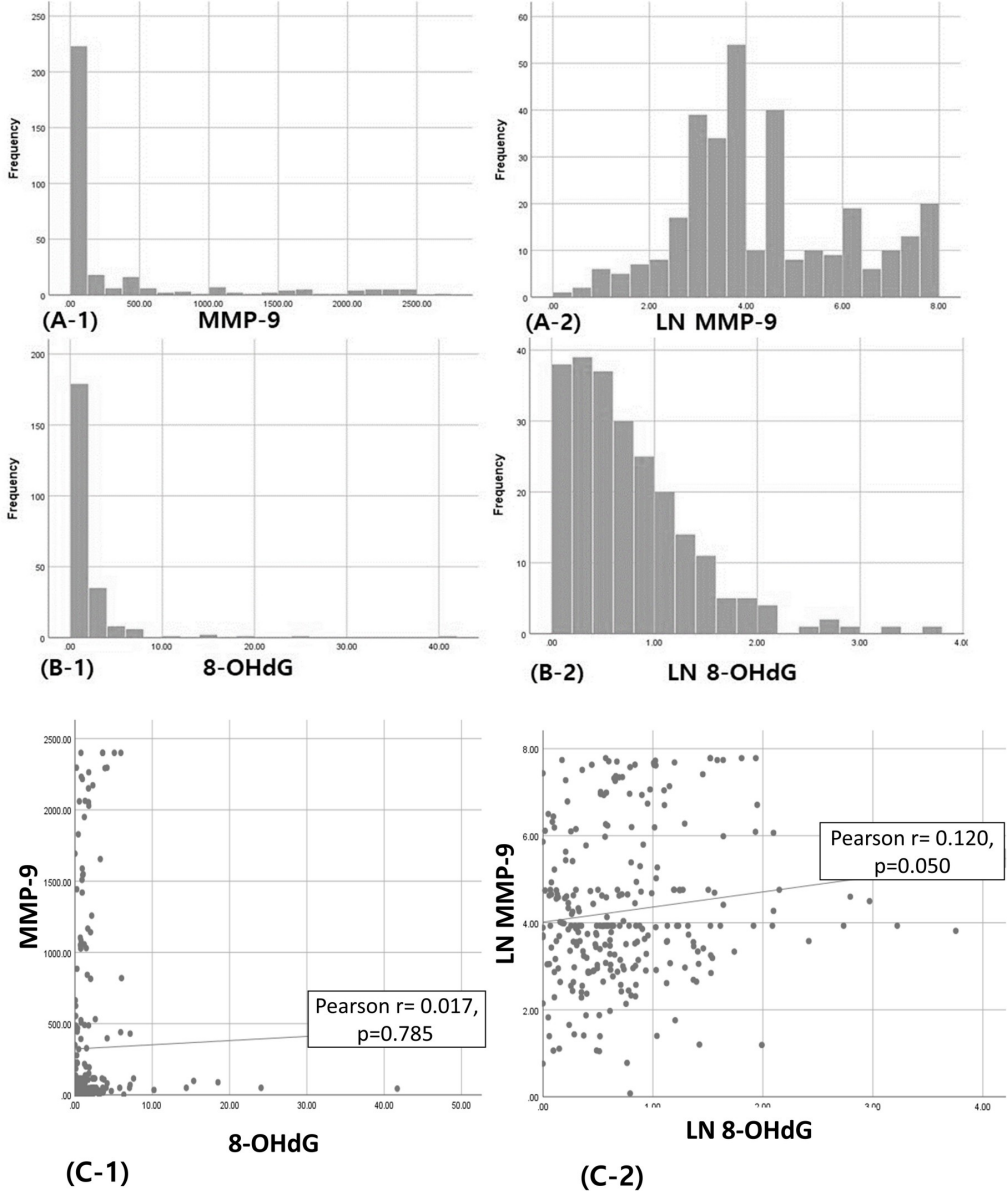
Distribution of salivary markers: MMP-9 (A-1), LN MMP-9 (A-2), 8-OHdG (B-1) and LN 8-OHdG (B-2); and Scatter plot for correlation between markers: MMP-9 versus 8-OHdG (C-1), LN MMP-9 versus LN 8-OHdG (C-2). Natural logarithm (LN): LN (1 + MMP-9) and LN (1 + 8-OHdG).

Although values of MMP-9 and 8-OHdG among participants were not normally distributed (Kolmogorov-Smirnov test, p<0.001 for MMP-9 and 8-OHdG), we applied non-parametric test based on median with 25 and 75 percentiles. For meeting the strict assumption of parametric analysis such as analysis of covariance (ANCOVA) for an adjusted mean and linear regression model for an adjusted association, the data of MMP-9 and 8-OHdG were transformed using natural logarithm (LN): LN (1 + MMP-9) and LN (1 + 8-OHdG) (Fig 1). However, natural log-transformed data of MMP-9 and 8-OHdG were also not normally distributed, but showed a better distribution. The correlation between MMP-9 and 8-OHdG was very low and non-significant (Pearson r = 0.017, p>0.05) (Fig 1). Moreover, the correlation between LN MMP-9 and LN 8-OHdG was a bit increased, but still non-significant in border line (Pearson r = 0.120, p = 0.050).

Kruskal-Wallis for median were applied to evaluate the difference in MMP-9 and 8-OHdG across the location and TNM staging of cancer cases. The difference in MMP-9 and 8-OHdG between OSCC cases and controls was tested by Mann-Whitney for median. ANCOVA was applied to evaluate the amount of salivary LN MMP-9 and salivary LN 8-OHdG adjusted for various covariates. Moreover, multivariable logistic regression model was applied to evaluate an association of salivary MMP-9 and salivary 8-OHdG with OSCC after controlling for various covariates. The algorithm of MMP-9 adjusted for covariates was developed using the results of multivariable logistic regression models.

For follow-up data analysis, Wilcoxon sign rank test was applied for between two adjacent follow-up period: operation versus 3 months; 3months versus 6 months; 6 months versus 9 months. The comparison across four follow-up measures was tested by using Friedman test for median.

The diagnostic ability of crude MMP-9 in saliva and adjusted MMP-9 algorithm was obtained through the receiver operating characteristic (ROC) curve. We applied non-parametric ROC curve. ROC curve produced the area under the curve (AUC) by numerical integration including sensitivity, specificity and C-statistics, which showed the diagnostic ability of the markers. Higher value of AUC that ranges from 0.5 to 1.0 reflects higher performance of the diagnostic ability. Thus, AUC value close to 1.0 suggests the perfect diagnostic ability.

All statistical analyses were conducted using SPSS software, version 25.0 (SPSS, Inc. Armonk, NY, USA). Statistical significance was set at p-value <0.05.

## Results

### Characteristics of participants

The total 318 participants for MMP-9 with mean age of 63.1 years was consisted of 58.5% of males, while total 234 participants for 8-OHdG with mean age of 63.6 years had 57.7% of males (Table 1). For MMP-9, our data between OSCC cases and controls showed that there was significant difference in alcohol intake, education level, physical activity, periodontitis, diabetes and hypercholesterolemia (p<0.05), while no difference in obesity and hypertension. The pattern was almost same for 8-OHdG except physical activity and diabetes which showed no difference between OSCC cases and controls.

### MMP-9 and 8-OHdG across OSCC location, TNM staging before surgery

For MMP-9, OSCC in the hard palate showed the highest value (1103.6 ng/ml for median), and OSCC in TNM stage 3 showed the highest value (1029.8 ng/ml for median) (Table 2), which were not statistically significant (P>0.05). For 8-OHdG, OSCC in the floor of the mouth showed the highest value (2.2 ng/ml for median), and OSCC in TNM stage 3 showed the highest value (1.5 ng/ml for median), which were not statistically significant, which were not statistically significant (P>0.05).

**Table 2:** Salivary MMP-9 and 8-OHdG of cancer cases according to the location, TNM staging, and follow-up after surgery.

	MMP-9 (ng/ml) (N = 106)		8-OHdG (ng/ml) (N = 78)	
	N (%)	Median (25; 75)	N (%)	Median (25; 75)
**Location**				
FOM	4 (3.8%)	882.74 (268.07; 2114.64)	3 (3.8%)	2.16 (0.80;—)
Tongue	23 (21.7%)	531.66 (202.28; 1141.91)	17 (21.8%)	1.23 (0.23; 1.90)
BM	18 (17.0%)	759.29 (333.16; 1461.88)	15 (19.2%)	0.93 (0.43; 1.38)
AR	48 (45.3%)	468.96 (177.45; 2166.92)	31 (39.7%)	1.33 (0.30; 3.26)
HP	5 (4.7%)	1103.57(525.40; 1921.66)	4 (5.1%)	0.46 (0.13; 0.75)
RT	8 (7.5%)	505.34 (269.67; 1420.86)	8 (10.3%)	0.89 (0.36; 1.65)
	*p-value	0.396	*p-value	0.242
**TNM staging**				
0	3 (2.8%)	395.11 (6.19;—)	2 (2.6%)	0.95 (0.84;—)
1	20 (18.9%)	332.49 (189.56; 1437.28)	17 (21.8%)	1.21 (0.18; 1.62)
2	35 (33.0%)	842.68 (397.53; 2029.04)	22 (28.2%)	0.96 (0.25; 2.16)
3	11 (10.4%)	1029.84(195.26; 1588.23)	7 (9.0%)	1.46 (0.69; 1.76)
4	37 (34.9%)	524.38 (272.78; 1525.62)	30 (38.5%)	0.86 (0.36; 1.85)
	*p-value	0.396	*p-value	0.952

G: 8-hydroxydeoxyguanosine; SD: Standard deviation; FOM: Floor of the mouth; BM: Buccal mucosa; AR: Alveolar ridge; HP: Hard palate; RT: Retromolar trigone.

*p-values were obtained from Kruskal-Wallis for median.

### Association of MMP-9 and 8-OHdG with OSCC

After controlling for various confounders, MMP-9 was significantly associated with OSCC: increase of 1ng/ml of MMP-9 was more likely by 5% to have OSCC (OR = 1.054, p<0.05) (Table 3). Among confounders, smoking, drinking, hypertension and hypercholesterolemia were significantly associated with OSCC (p<0.05). However, OSCC was not associated with 8-OHdG (p>0.05).

**Table 3:** Adjusted association of MMP-9 and 8-OHdG with oral squamous cell carcinoma (OSCC) using multivariable logistic regression models.

Variable	LN MMP-9 (N = 318)			LN 8-OHdG (N = 234)		
	Beta	OR (95% CI)	p- value	Beta	OR (95% CI)	p- value
MMP-9 (ng/ml)	**0.052**	**1.054 (1.032–1.076)**	**<0.001**			
8-OHdG (ng/ml)				-0.082	0.921 (0.811–1.046)	0.204
Age	0.009	1.009 (0.911–1.118)	0.859	0.037	1.037 (0.994–1.082)	0.089
Sex	1.87	6.486 (0.69–60.969)	0.102	0.506	1.659 (0.695–3.959)	0.254
Smoking	**2.433**	**11.397 (1.256–103.394)**	**0.031**	0.508	1.663 (0.694–3.981)	0.254
Drinking	**-2.517**	**0.081 (0.014–0.472)**	**0.005**	**-1.100**	**0.333 (0.169–0.656)**	**0.001**
Education	1.53	4.618 (0.776–27.501)	0.093	**1.778**	**5.915 (2.778–12.598)**	**<0.001**
Physical activity	-0.447	0.64 (0.145–2.813)	0.554	**-0.843**	**0.43 (0.219–0.846)**	**0.015**
Periodontitis	0.825	2.282 (0.722–7.216)	0.16	0.851	2.342 (1.377–3.983)	0.002
Obesity	0.512	1.669 (0.39–7.131)	0.49	-0.14	0.869 (0.448–1.688)	0.679
Diabetes	-0.302	0.739 (0.084–6.531)	0.786	0.516	1.675 (0.742–3.782)	0.215
Hypertension	**2.741**	**15.499 (2.221–108.182)**	**0.006**	0.335	1.398 (0.702–2.784)	0.34
Hypercholesterolemia	**3.434**	**31.004 (3.977–241.695**	**0.001**	**1.794**	**6.013 (2.088–17.315)**	**0.001**
Constant	-14.936		0.023	-5.666		0.019

Odds Ratio (OR) were adjusted for all variables except the designated variable in this Table.

Bold denotes statistical significance at p<0.05.

### Crude and Adjusted MMP-9 and 8-OHdG values between OSCC cases and controls

The crude median value of salivary MMP-9 (ng/ml) in OSCC cases was significantly higher by 15 times than that in controls (p<0.05) (Table 4). Moreover, after adjustments of confounders, adjusted original mean value of salivary MMP-9 in OSCC was still significantly higher by 17 times than that in controls (p<0.05). However, salivary 8-OHdG (ng/ml) between OSCC cases and controls did not show any significant differences in crude median values (p>0.05) nor in adjusted mean value (p>0.05).

**Table 4:** Comparison of MMP-9 and 8-OHdG values between oral squamous cell carcinoma (OSCC) cases and controls in pre-surgery.

Pre-surgery	OSCC		*p*-value
	Control	Case	
**MMP-9 (ng/ml)**	N = 212	N = 106	
Crude*, median (25; 75)	**34.14 (17.05; 50.00)**	**522 (215; 1635)**	**< 0.001**
Adjusted^†^, mean LN ± SE LN	**3.41 ± 0.08**	**6.24 ± 0.11**	**< 0.001**
(original value)	**(29.27 ± 0.08)**	**(511.86 ± 0.12)**	
**8-OHdG (ng/ml)**	N = 156	N = 78	
Crude*, median (25; 75)	0.79 (0.35; 1.79)	0.95 (0.31; 1.77)	0.558
Adjusted^†^, mean LN ± SE LN	0.78 ± 0.05	0.73 ± 0.08	0.605
(original value)	(1.18 ± 0.05)	(1.08 ± 0.08)	

Bold denotes statistical significance at p<0.05.

SE: standard error.

*Crude values were obtained from Mann-Whitney for median.

^†^Adjusted values of LN were obtained from analysis of covariance (ANCOVA) in general linear model with covariates of age, sex, smoking, alcohol intake, education level, physical activity, periodontitis, obesity, diabetes, hypertension, and hypercholesterolemia. Natural logarithm (LN): LN (1 + MMP-9) and LN (1 + 8-OHdG).

### Changes in MMP-9 before and after surgery

**Fig 2:**
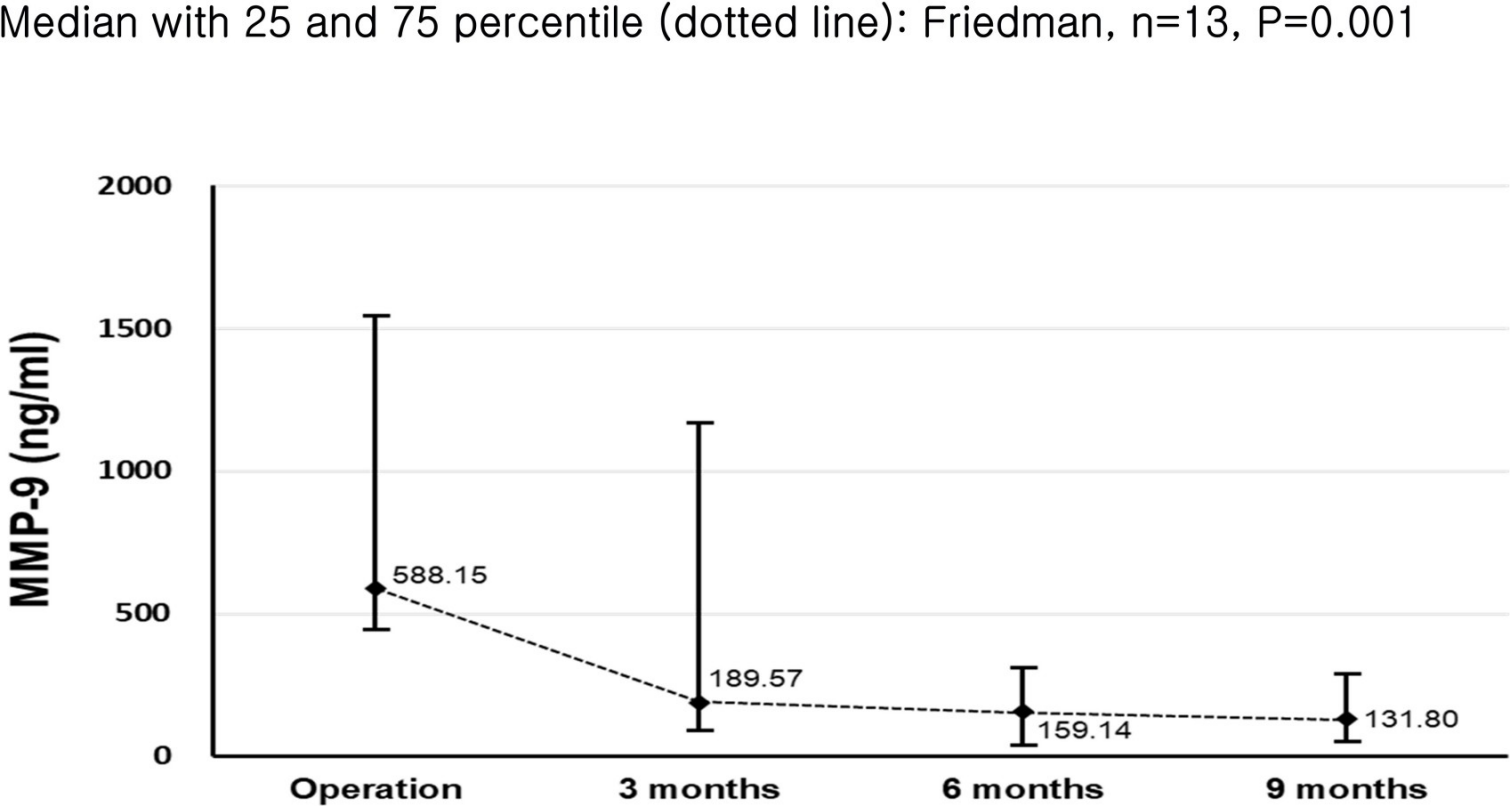
Changes in MMP-9 of median (dotted line) across 3-, 6-, 9-month after surgery. Friedman test was applied for statistical significance.

After tumor surgery, 9 months follow-up values of MMP-9 (ng/ml) were decreased by 80% from the value in operation (p<0.05) (Table 5, Fig 2). However, the reduction in MMP-9 was significant only in three months follow-up by 65% (p<0.05). The value of MMP-9 was continuously decreased in six and nine months, which was not statistically significant between two adjacent groups after three months (p>0.05). Moreover, the value of MMP-9 in nine months after surgery was still higher than MMP-9 value of controls (p<0.05): median of 34.1 for control versus 102.6 for nine months after surgery.

**Table 5:** Changes in MMP-9 values in OSCC cases across follow-up time in post- surgery.

Post-surgery	Follow -up	MMP-9 (ng/ml)	N	p-value^‡^
	N (%)	median (25;75)		
**Operation**	106 (100.0%)	**522.19** (**215.11; 1635.22)**	50	**<0.001**
**3 months**	50 (47.17%)	**185.74 (60.3; 516.53)**		
**6 months**	44 (41.51%)	209.83 (68.78; 447.80)	27	0.545
**9 months**	42 (39.62%)	102.60 (13.14; 279.69)	23	0.833

Bold denotes statistical significance at p<0.05.

^‡^ *p*-values were obtained from Wilcoxon sign rank sum for median.

### Screening ability of MMP-9

**Fig 3:**
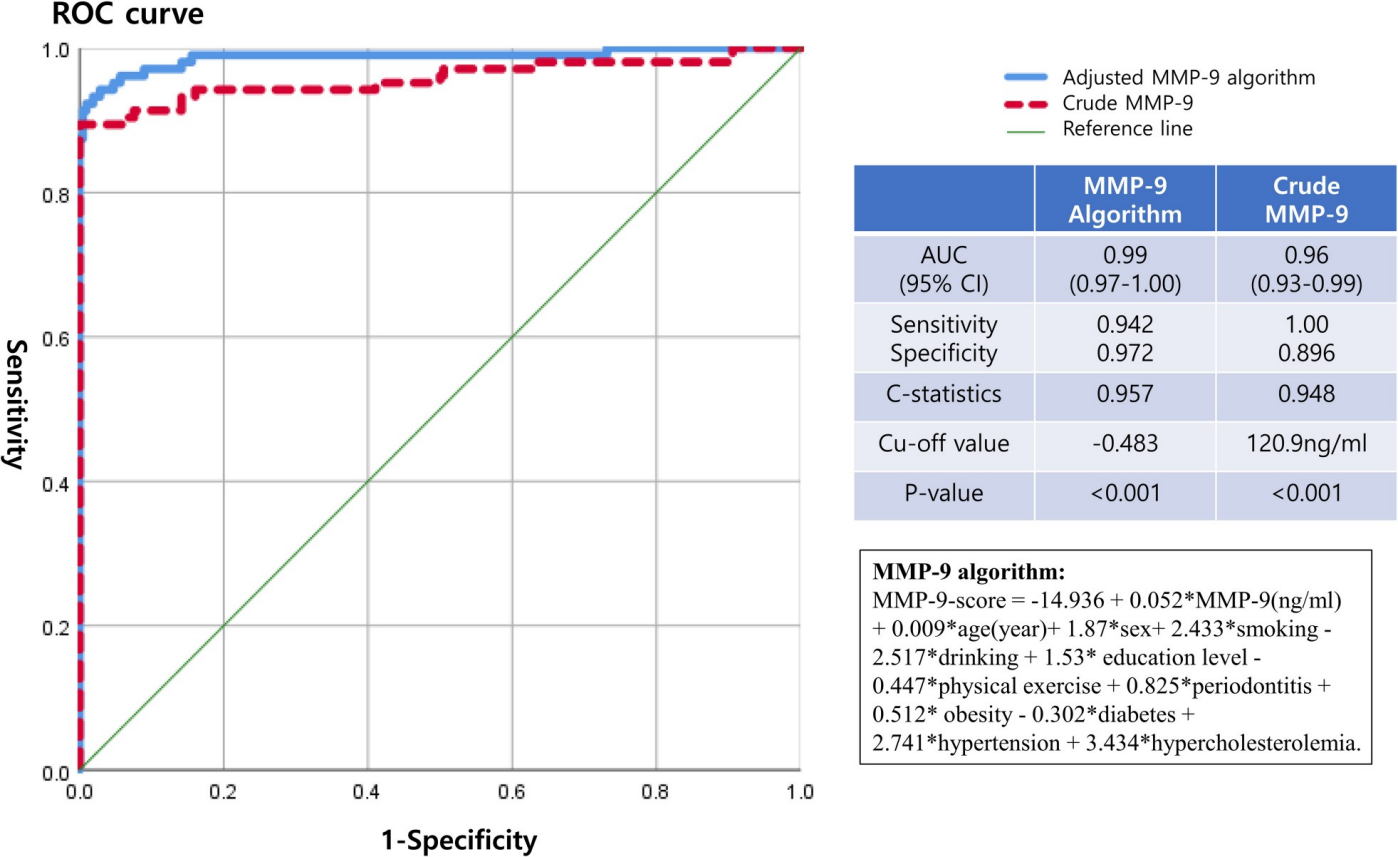
Receiver operating characteristic curve of crude MMP-9 and MMP-9 algorithm for diagnostic ability of oral squamous cell carcinoma.

A ROC curve analysis was performed and AUC was calculated for crude salivary MMP-9 and adjusted MMP-9 using algorithm including salivary MMP-9 and all covariates. According to the logistic regression model for MMP-9 (Table 3), MMP-9 algorithm was MMP-9-score = -14.936 + 0.052*MMP-9(ng/ml) + 0.009*age(year)+ 1.87*sex+ 2.433*smoking—2.517*drinking + 1.53* education level -0.447*physical exercise + 0.825*periodontitis + 0.512* obesity—0.302*diabetes + 2.741*hypertension + 3.434*hypercholesterolemia. In our study, the AUC value of crude MMP-9 (p<0.001) was 0.96 (100% specificity, 89.6% sensitivity, C-statistics of 0.948) with 95% CI of 0.93–0.99 under the cut off value of 120.9 ng/ml in MMP-9 (Fig 3). The AUC value of adjusted MMP-9 algorithm was 0.99 (94.2% specificity, 97.2% sensitivity, C-statistics of 0.957) with 95% CI of 0.97–1.00. Our data suggested that crude MMP-9 of 120.9 ng/ml was the critical value as a potential biomarker for screening OSCC risk.

## Discussion

The results of our OSCC cases and age, sex, and smoking-match controls study indicate that salivary biomarker MMP-9 demonstrate high discriminatory power for screening OSCC. Our data confirmed that salivary MMP-9 was associated with oral cancer including OSCC [25]. In our study, MMP-9 did not show significant difference in tumor location and in TNM staging, which suggested that MMP-9 could not be location and/or staging specific but universal screening tool for OSCC. Especially, the fact that any healthy control participants did not show the salivary MMP-9 value above the reported cut-off value (120.9 ng/ml) is encouraging. Since our data showed that both crude MMP-9 and adjusted MMP-9 algorithm had the similarly high diagnostic ability (C-statistics: 0.95 for crude MMP-9 and 0.96 for adjusted MMP-9 algorithm), crude MMP-9 could be selected as the appropriate tool for the screening of OSCC. Moreover, MMP-9 decreased dramatically after surgery, which means that MMP-9 could be a prognostic marker for OSCC. To the best of our knowledge, this is the first evidence that surgical removal of OSCC reduced the level of salivary MMP-9, which showed a prognostic ability of salivary MMP-9 on pre- and post- surgery of OSCC. Overall, salivary MMP-9 is an excellent diagnostic and prognostic marker for OSCC.

This study has some advantages. Firstly, OSCC cases were diagnosed using CT, MRI, PET-CT and biopsy in the SNU dental hospital and periodontitis was assessed by panoramic radiograph, which reduced the misclassification bias. Secondly, age, sex and smoking-matched controls were from the community cohort, which reduced the selection bias due to hospital control. Thirdly, we traced cancer patient prospectively after surgery, which clarify the temporality of prognostic ability of MMP-9 for OSCC. Finally, we considered well-known risk factors such as alcohol intake, education level, physical activity, obesity, diabetes, hypertension and hypercholesterolemia as confounders, which reduce the over-estimation due to less adjustment confounders.

The mechanism of MMP-9 is to degrade type-IV collagen, a major component of basement membrane, as well as other types of collagens such as type V, VII and X, and elastin, and fibronectin.

MMP-9 is one of the major gelatinase-degrading enzymes, which is mainly responsible for degradation of matrix proteins in inflamed tissue during gingivitis and adult periodontitis [25,26]. Moreover, MMP-9 may be an indicator for early diagnosis of oral cancer (OC) [27]. MMP-9 was reported as a useful marker for tumor metastases and for prognosis in patients including early-stage OSCC [28], another study correlated MMP-9 expression with tumor invasion and lymph node involvement in oral SCC [29]. There were also reports that MMP-9 expression did not correlate with tumor and lymph node stages but correlated as a prognostic factor for unfavorable survival in head and neck carcinoma [30,31]. Our pre-surgery data showed that neither salivary MMP-9 nor 8-OHdG had any significant difference according to tumor location and TNM staging. Therefore, more studies about impact of MMP-9 on TNM staging and prognosis must be indicated to clarify the difference.

Our post-surgery data showed that the level of salivary MMP-9 after surgery was dramatically decreased for 3 months and gradually decreased for 3–9 months, while the level of MMP-9 in 9 months after surgery was still higher (102 ng/ml) than the controls (34 ng/ml). Other remaining inflammation such as periodontitis could be contributing factors on these results. Protracted oropharyngeal mucosal inflammation from postoperative radiation therapy or concurrent chemoradiation therapy which begins usually around 1 month of surgery and takes about 2 months until completion, may also result in MMP-9 elevation. Reduced salivary flow rate from chemotherapy, removal of salivary gland, and/or radiation therapy to the head and neck region may contribute to prolonged elevation of MMP-9 level up to about 1 year post-operatively. Cervical lymphatic metastases or field cancerization seems to be the more plausible cause of high MMP-9 against control, because median recurrence time from initial surgery is usually 7.5 months (0.9–143.9 months) [32]. Field cancerization is often used to describe the development of abnormal tissues around a tumorigenic area, resulting into an oral multifocal cancer in individual sites, which later coalesce and create atypical areas, even after complete surgical removal [33]. Due to the small follow-up patients in 9 months post-surgery (n = 13), our data did not show whether and when the level of MMP-9 reduced to the level of controls. Hence, further studies with more follow-up patients and influencing factors mentioned as above should be indicated to clarify it.

Early detection of disease is deemed paramount to decrease patient mortality and morbidity rates. Salivary biomarkers mirror patient’s health status by utilizing the highly sensitive assays, which leads to early detection of OSCC [34]. Saliva has rich source of protein biomarkers [35]. Salivary biomarkers are capable of distinguishing healthy from non-healthy patients [36]. More than 100 salivary biomarkers have been reviewed for detecting OSCC in 2014 [37]. Collecting saliva is non-invasive, easily reproducible, accessible, not time-consuming, inexpensive. Hence, saliva may be used for mass screening of large population [38]. Combinational use of validated salivary biomarkers will accelerate highly sensitive screening tools in detecting early stage OSCC. Saliva would open an innovative frontier in high-quality healthcare using highly sensitive detection tools. A salivary MMP-9 based point-of-care test could allow people to have a routine real-time monitoring system.

Our findings did not show any significant association of DNA damage marker 8-OHdG with OSCC. The involvement of free radicals in cancer development has been studied for three decades and 8-OHdG has been a marker of oxidative stress to DNA. In counter to our results, several works explore the levels of oxidative stress in patients with increased risk of oral cancer [13,39]. Especially, 8-OHdG could provide a starting point as an oral biomarker for monitoring in avoiding malignant transformation [12]. Hence, further studies are indicated to clarify the differences.

The limitations of this study were as follows. Firstly, saliva was not collected in the same time frame for cases and controls, which might decrease the salivary protein amount in controls recruited some years earlier than cases. Secondly, this study was not predictive but diagnostic. Hence, the future predictive research should be indicated. It must focus on the early assessment of such salivary biomarkers among risk group in advance of OSCC occurrence. Finally, interaction between tobacco and alcohol use [40] should be considered in the future for this type of analysis. Despite these limitations, our data was suitable to evaluate the association and screening ability of salivary MMP-9 for OSCC.

## Conclusion

Our findings indicate that salivary MMP-9 could be a screening and prognostic biomarker for OSCC. Early detection of OSCC using salivary MMP-9 could be deemed paramount to decrease patient mortality and morbidity rates due to OSCC.

## Supporting information

S1 Checklist STROBE statement—checklist of items that should be included in reports of observational studies. (DOCX)

S1 File (SAV)
