# Supplementary material for: Diagnostic and prognostic ability of salivary MMP-9 for oral squamous cell carcinoma: A pre-/post-surgery case and matched control study
Source: PLoS One. 2021 Mar 18;16(3):e0248167. doi: 10.1371/journal.pone.0248167 (PMC7971541; doi:10.1371/journal.pone.0248167)
Supplement: S1 Checklist — (DOCX) [file pone.0248167.s001.docx]

STROBE Statement—checklist of items that should be included in reports of observational studies

|  | Item No. | Recommendation | Page  No. | Relevant text from manuscript |
| --- | --- | --- | --- | --- |
| **Title and abstract** | 1 | (*a*) Indicate the study’s design with a commonly used term in the title or the abstract | 1 in the title | Case-control study including follow-up cases |
|  |  | (*b*) Provide in the abstract an informative and balanced summary of what was done and what was found | 2 in abstract | MMP-9 as universal screening and prognostic marker for OSCC |
| Introduction | | | |  |
| Background/rationale | 2 | Explain the scientific background and rationale for the investigation being reported | 3-4 in introduction | -association of salivary MMP-9 and 8-OHdG  -small number of participants and no/insufficient adjustments |
| Objectives | 3 | State specific objectives, including any prespecified hypotheses | 4 in introduction | -salivary biomarkers are associated with OSCC |
| Methods | | | |  |
| Study design | 4 | Present key elements of study design early in the paper | 4-5 in materials and methods | case-control matched to age, sex and smoking |
| Setting | 5 | Describe the setting, locations, and relevant dates, including periods of recruitment, exposure, follow-up, and data collection | 4-5 in materials and methods | Cases from SNUDH, 2015-2017  Control form Yangpyeong cohort, 2010-2014 |
| Participants | 6 | (*a*) *Cohort study*—Give the eligibility criteria, and the sources and methods of selection of participants. Describe methods of follow-up  *Case-control study*—Give the eligibility criteria, and the sources and methods of case ascertainment and control selection. Give the rationale for the choice of cases and controls  *Cross-sectional study*—Give the eligibility criteria, and the sources and methods of selection of participants |  |  |
|  |  | (*b*) *Cohort study*—For matched studies, give matching criteria and number of exposed and unexposed  *Case-control study*—For matched studies, give matching criteria and the number of controls per case | 4-5 in materials and methods | Age, sex and smoking, 1:2 ratio  As a result, 318 participants (106 cases and 212 controls) for MMP-9 and 234 participants (78 cases and 156 controls) for 8-OHdG |
| Variables | 7 | Clearly define all outcomes, exposures, predictors, potential confounders, and effect modifiers. Give diagnostic criteria, if applicable | 5-7 in materials and methods | Effect modifier: age, sex, smoking, tooth loss  Confounders: age, sex, smoking, alcohol intake, education level, physical activity, obesity, diabetes, hypertension and hypercholesterolemia  Diagnostic criteria: Periodontitis-5th European guideline; OSCC-location and TMN stage |
| Data sources/ measurement | 8* | For each variable of interest, give sources of data and details of methods of assessment (measurement). Describe comparability of assessment methods if there is more than one group | 5-8 in materials and methods | Periodontitis: panoramic radiograph  OSCC: biopsy and enhanced CT  MMP-9 and 8-OHdG : ELISA |
| Bias | 9 | Describe any efforts to address potential sources of bias | 13 in discussion | Saliva collection time frame  Retrospective study |
| Study size | 10 | Explain how the study size was arrived at | 4 in materials and methods | Sample size estimation |
| Quantitative variables | 11 | Explain how quantitative variables were handled in the analyses. If applicable, describe which groupings were chosen and why | 6-8 in materials and -methods | MMP-9 and 8-OHdg by ELISA test  Blood pressure, cholesterol, BMI, glucose were dichotomized. |
| Statistical methods | 12 | (*a*) Describe all statistical methods, including those used to control for confounding | 8-9 in materials and methods (statistical analyses) | Chi-square for categorical variables  T-test, ANOVA for continuous variables  Analysis of covariance for adjustment  Paired-T test and repeated measure ANOVA for follow-up period.  Receiver operating characteristics curve for screening ability. |
|  |  | (*b*) Describe any methods used to examine subgroups and interactions | N/A |  |
|  |  | (*c*) Explain how missing data were addressed | 5 in materials and methods | Excluded – not enough salivary sample |
|  |  | (*d*) *Cohort study*—If applicable, explain how loss to follow-up was addressed  *Case-control study*—If applicable, explain how matching of cases and controls was addressed  *Cross-sectional study*—If applicable, describe analytical methods taking account of sampling strategy | 4-5 in materials and methods (study design and sample size estimation) | Case-control: matched to age, sex and smoking with a ratio of 1:2 |
|  |  | (*e*) Describe any sensitivity analyses | N/A |  |
| Results | | | | |
| Participants | 13* | (a) Report numbers of individuals at each stage of study—eg numbers potentially eligible, examined for eligibility, confirmed eligible, included in the study, completing follow-up, and analysed | 4-5 in materials and methods  9 in results  15 in Table 1 | 318 participants (106 cases and 212 controls) for MMP-9 and 234 participants (78 cases and 156 controls) for 8-OHdG |
|  |  | (b) Give reasons for non-participation at each stage | 6 in materials and methods | Excluded |
|  |  | (c) Consider use of a flow diagram | N/A | N/A |
| Descriptive data | 14* | (a) Give characteristics of study participants (eg demographic, clinical, social) and information on exposures and potential confounders | 4-5 in materials and methods  9 in results  15 in Table 1 | Age, sex, smoking, alcohol intake, education level, physical activity, obesity, diabetes, hypertension and hypercholesterolemia |
|  |  | (b) Indicate number of participants with missing data for each variable of interest | N/A | N/A |
|  |  | (c) *Cohort study*—Summarise follow-up time (eg, average and total amount) | N/A | N/A |
| Outcome data | 15* | *Cohort study*—Report numbers of outcome events or summary measures over time |  |  |
|  |  | *Case-control study—*Report numbers in each exposure category, or summary measures of exposure | Table 1 | Outcome: OSCC  Exposure: MMP-9 and 8-OHdG |
|  |  | *Cross-sectional study—*Report numbers of outcome events or summary measures |  |  |
| Main results | 16 | (*a*) Give unadjusted estimates and, if applicable, confounder-adjusted estimates and their precision (eg, 95% confidence interval). Make clear which confounders were adjusted for and why they were included | 10-11 in results  Tables 2-3 | Well known factors: age, sex, smoking, alcohol intake, education level, physical activity, obesity, diabetes, hypertension and hypercholesterolemia |
|  |  | (*b*) Report category boundaries when continuous variables were categorized | 6-7 in materials and methods | For age, obesity (BMI), diabetes (FPG), hypertenstion (SBP and DBP) and hypercholesterolemia (FTC). |
|  |  | (*c*) If relevant, consider translating estimates of relative risk into absolute risk for a meaningful time period | N/A | N/A |
| Other analyses | 17 | Report other analyses done—eg analyses of subgroups and interactions, and sensitivity analyses | 10-11 in results  Fig 2-3 | Follow up period: paired-T and repeated measure  ANOVA, ROC for screening ability |
| Discussion | | | | |
| Key results | 18 | Summarise key results with reference to study objectives | 11-13 in discussion | Salivary biomarker MMP-9 demonstrate high discriminatory power for screening and prognosis of OSCC |
| Limitations | 19 | Discuss limitations of the study, taking into account sources of potential bias or imprecision. Discuss both direction and magnitude of any potential bias | 13 in discussion | Saliva collection time frame and retrospectively studied |
| Interpretation | 20 | Give a cautious overall interpretation of results considering objectives, limitations, multiplicity of analyses, results from similar studies, and other relevant evidence | 11-13 in discussion | Our results were compared and supported with previous studies. |
| Generalisability | 21 | Discuss the generalisability (external validity) of the study results | 12 in discussion | No selection bias |
| Other information |  | | | |
| Funding | 22 | Give the source of funding and the role of the funders for the present study and, if applicable, for the original study on which the present article is based | 14 in acknowledgements (funding source) | Ministry of science and ICT, Korea (NRF-2017M3A9B6062984) |

*Give information separately for cases and controls in case-control studies and, if applicable, for exposed and unexposed groups in cohort and cross-sectional studies.

**Note:** An Explanation and Elaboration article discusses each checklist item and gives methodological background and published examples of transparent reporting. The STROBE checklist is best used in conjunction with this article (freely available on the Web sites of PLoS Medicine at http://www.plosmedicine.org/, Annals of Internal Medicine at http://www.annals.org/, and Epidemiology at http://www.epidem.com/). Information on the STROBE Initiative is available at www.strobe-statement.org.
